# An update on COVID-19: SARS-CoV-2 variants, antiviral drugs, and vaccines

**DOI:** 10.1016/j.heliyon.2023.e13952

**Published:** 2023-02-23

**Authors:** Varghese Edwin Hillary, Stanislaus Antony Ceasar

**Affiliations:** Department of Biosciences, Rajagiri College of Social Sciences, Cochin, 683 104, Kerala, India

**Keywords:** Antiviral drugs, COVID-19, SARS-CoV-2, SARS-CoV-2 variants, Vaccines, SARS-CoV-2, Severe acute respiratory syndrome coronavirus 2, COVID-19, Coronavirus infectious disease-2019, WHO, World Health Organization, ACE2, Angiotensin-converting enzyme 2, RBD, Receptor-binding domain, VOC, Variants of Concern, VOI, Variants of Interests, FDA, Food and Drug Administration, NIH, National Institutes of Health, EUA, Emergency Use Authorization

## Abstract

Severe acute respiratory syndrome coronavirus 2 (SARS-CoV-2) is a highly contagious and pathogenic virus that first appeared in late December 2019. This SARS-CoV-2 causes an infection of an acute respiratory disease called “coronavirus infectious disease-2019 (COVID-19). The World Health Organization (WHO) declared this SARS-CoV-2 outbreak a great pandemic on March 11, 2020. As of January 31, 2023, SARS-CoV-2 recorded more than 67 million cases and over 6 million deaths. Recently, novel mutated variants of SARS-CoV are also creating a serious health concern worldwide, and the future novel variant is still mysterious. As infection cases of SARS-CoV-2 are increasing daily, scientists are trying to combat the disease using numerous antiviral drugs and vaccines against SARS-CoV-2. To our knowledge, this is the first comprehensive review that summarized the dynamic nature of SARS-CoV-2 transmission, SARS-CoV-2 variants (a variant of concern and variant of interest), antiviral drugs and vaccines utilized against SARS-CoV-2 at a glance. Hopefully, this review will enable the researcher to gain knowledge on SARS-CoV-2 variants and vaccines, which will also pave the way to identify efficient novel vaccines against forthcoming SARS-CoV-2 strains.

## Highlights

• SARS-CoV-2 is a highly contagious and pathogenic virus.
• This study discussed the details of SARS-CoV-2 variants.
• AntiViral drugs used against SARS-CoV-2 are briefly summarized.
• Vaccines developed against SARS-CoV-2 are discussed.
• This study will help the researchers to gain knowledge on SARS-CoV-2 variants, antiviral drugs, and vaccines.

## 1 Introduction

At the end of 2019, World Health Organization (WHO) warned of cases of infectious diseases (unknown viruses) emerging in Wuhan city, Hubei province, central China [1]. These respiratory illness symptoms were like viral pneumonia and manifested as cough, dyspnea, and fever [1]. Soon after, scientists isolated this unknown virus, performed high throughput sequencing in December 2019, and identified a new Beta type of coronavirus (β-CoV) strain [2,3]. This novel CoV strain was hereditarily parallel to the existing bat severe acute respiratory syndrome (SARS-SL-CoVZC45 and SL-CoVZXC21 strains with 88% homology), SARS-CoV-1 (79.5% homology), and Middle East respiratory syndrome (MERS) (50% homology) coronaviruses [3,4]. Due to its genetic similarity with previous CoV strains, the International Committee on Taxonomy of Viruses (ICTV) and WHO officially declared this novel β-CoV strain as a new type of SARS-CoV-2 virus.

SARS-CoV-2 directly infects the upper respiratory tract (sinus, nose, and throat) and lower respiratory tract (windpipe and lungs) of humans. Before SARS-CoV-2, there were other CoV family strains like human coronavirus NL63 (hCoV-NL63), hCoV-229E, hCoV-OC43, hCoV-HKU1, hCoV-NL63, which infect humans and cause major health problems [[5], [6], [7], [8]]. Patients with SARS-CoV-2 infection typically present with acute respiratory illness and symptoms, including headache, nausea, cough, vomiting, diarrhea, and fever [3,9]. The severity of SARS-CoV-2 infection is mild in most people, but some elders and people with other health issues face major problems like multi-organ dysfunction and acute respiratory distress syndrome (ARDS). The disease severity of SARS-CoV-2 also differs from asymptomatic to symptomatic persons [3,9]. As of 31st January 2023, 67 million cases have been reported and the death toll had exceeded 6.8 million across the world.

The high case-to-fatality ratio and frequent outbreaks of SARS-CoV-2 triggered calls to develop effective drugs or vaccines. Therefore, scientists have developed emergency vaccines as the chief mitigation strategy to fight SARS-CoV-2 worldwide and are still working on developing vaccines and medicine for the new types of SARS-CoV-2 variants. Since SARS-CoV-2 variants increases the morbidity and mortality rates, decrease susceptibility to vaccines, increase transmissibility, skill to infect vaccinated hosts, etc. Therefore, this review article primarily provides a bird's-eye view on the SARS-CoV-2 variants. Secondly, it discusses the effectiveness of antiviral medicines and vaccines work against SARS-CoV-2. We believe this article would help researchers to gain knowledge and develop effective control measures against SARS-CoV-2 and their variants in the future.

## 2 Evolution of SARS-CoV-2

The first human CoV (B814) was identified in 1965 (England), which directly infects the upper respiratory tract of humans. In 2002, an unknown coronavirus (β genera) from bats emerged and infected humans via the intermediary host of palm civet cats (*Paguma larvata*) in China's Guangdong province. This virus was termed SARS-CoV, which affected 8422 people in China and Hong Kong and caused 900 deaths with a mortality of 11% [10]. Following this SARS-CoV, two additional CoVs such as HCoV-NL63 (in the year 2004) [11] and HCoV–KHU1 (in the year 2005) [12], were identified in horseshoe bats (genus *Rhinolophus*) in the Netherlands and Hong Kong, respectively [[13], [14], [15], [16], [17], [18]]. According to the ICTV report, the strains found only in *Rhinolophus* bats in China, Europe, and Southeast Asia are SARS-CoV variants. In 2019, a novel CoV called SARS-CoV-2 was identified in Wuhan, China, and infected humans via the intermediary host of animals. This novel SARS-CoV-2 has rapidly spread all over the countries as a world threat. On March 11, 2020, the WHO declared SARS-CoV-2 as a pandemic following the 1918 Spanish flu (H1N1) that caused over 50 million human deaths [19], the 1957 Asian flu (H2N2) that caused over 1.5 million human deaths [20], 1968 Hong Kong flu (H3N2) that caused over 1 million human deaths [21], and 2009 pandemic flu (H1N1), which caused 3,00,000 human deaths [22].

## 3 Structure of SARS-CoV-2

**Fig. 1:**
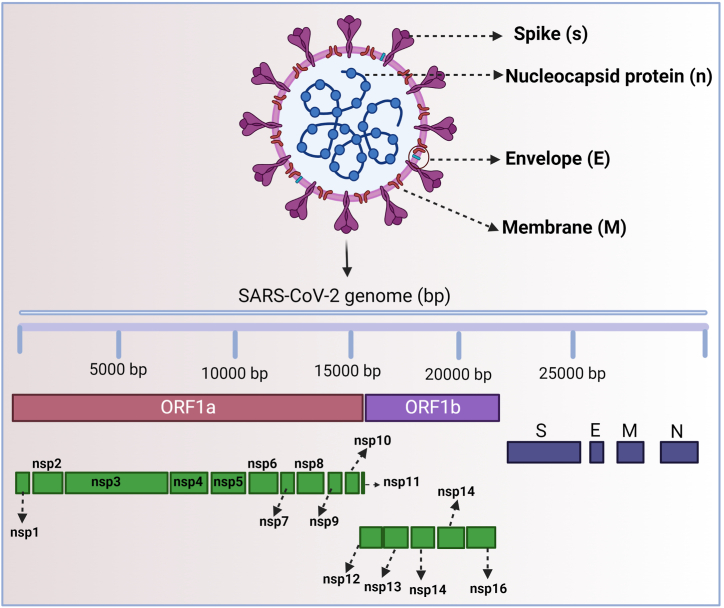
Structure of severe acute respiratory syndrome coronavirus-2 (SARS-CoV-2).

SARS-CoV-2 is the causative agent of COVID-19, which escalated into a global pandemic in 2020. SARS-CoV-2 is an enclosed, spherical, non-segmented, positive single-stranded RNA (ssRNA) with a 30 kbp genome belonging to the family Coronaviridae. SARS-CoV-2 is shielded by the helical capsid made by the nucleocapsid (N) protein and enclosed by an envelope protein (E) [23,24]. The structure of the SARS-CoV-2 protein contains E and membrane (M) proteins that help in virus assembly, and spike (S) protein allows the virus to enter into the hosts [16,25]. Among these E, M, and S proteins, the S protein size is too large (180–200 kDa) and appears like a crown [8]. Due to its “crown-like” spike appearance, the researchers named the novel SARS-CoV-2 strain a coronavirus (Fig. 1) (the Latin word corona means “crown”) [16,25].

In addition to structural proteins (S, E, M, and N), SARS-CoV-2 also contains non-structural proteins (nsps 1 to 16), which are involved in various functions. Nsp1 helps in the processing and replication of viral RNA. Nsp2 regulates the survival signaling pathway of the host cell. Nsp3 splits the translated protein (not completely revealed). Nsp4 contains transmembrane domain 2 (TM2) that alters ER membranes. Nsp5 plays a major role in the replication of polyproteins. Nsp6 functions as a transmembrane. Both nsp7 and nsp8 proteins significantly improve the arrangement of nsp12 and template-primer RNA. Nsp9 serves as an ssRNA binding protein, but its complete mechanisms are still unclear. The nsp10 involves in viral mRNA cap methylation. The nsp11 is an intrinsically disordered protein (mechanism still unclear) and nsp12 contains RNA-dependent RNA polymerase (RdRp), which is a vital component of virus replication and transcription. The nsp13 interacts with ATP and the zinc-binding domains and involves in RNA transcription and virus replication. Nsp14 is an exoribonuclease (EXoN) that majorly involves in proof reading during viral RNA synthesis. Nsp15 has an endoribonuclease activity, which is Mn (2+) dependent. The nsp16 is a 2′-O-ribose methyl transferase and it interacts with the U1 and U2 snRNAs' mRNA recognition domains during SARS-CoV-2 infection to prevent mRNA splicing (Fig. 1).

## 4 Routes of SARS-CoV-2 entry

SARS-CoV-2 entry into host cells is a vital cause of viral contagion and pathogenesis. Like all CoVs, the SARS-CoV-2 also utilizes the S glycoprotein to enter into the host cells [26]. This S glycoprotein of SARS-CoV-2 cleaves into two subunits, S1 and S2, in the infected cells [26]. SARS-CoV-2 S1 subunit contains a receptor-binding domain (RBD) and N-terminal domain (NTD) that binds to the angiotensin-converting enzyme 2 (ACE2) receptor, and SARS-CoV-2 S2 subunit attach to the virus particles to enter the host cell membrane [26].

**Fig. 2:**
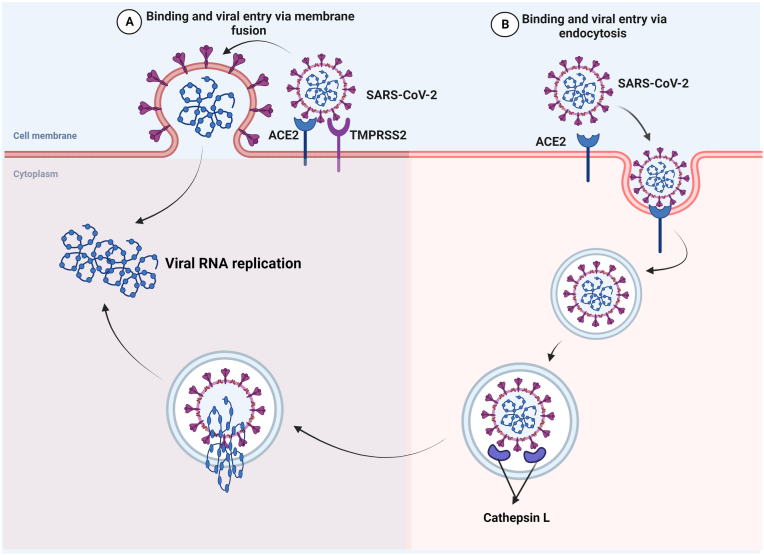
**Routes of severe acute respiratory syndrome coronavirus-2 (SARS-CoV-2) entry**: The SARS-CoV-2 enters target cells either by (A) membrane fusion or by (B) receptor-mediated endocytosis. (A) In membrane fusion, the S protein is mediated by transmembrane peptidase/serine subfamily member 2 (TMPRSS2), resulting in fusion of the viral membrane with the plasma membrane. (B) Binding of SARS-CoV-2 to ACE2 receptor leads to virus uptake into endosomes. The spike (S) glycoprotein is activated by cysteine peptidase cathepsin L in endo-lysosomes. In both way (A or B), the RNA genetic material of the virus will enter and starts RNA replication. (B) Binding of SARS-CoV-2 to ACE2 receptor leads to virus uptake into endocytosis. The spike (S) glycoprotein is activated by cysteine peptidase cathepsin L in endo-lysosomes. In both way (A or B), the RNA genetic material of the virus will enter and starts RNA replication.

**Fig. 3:**
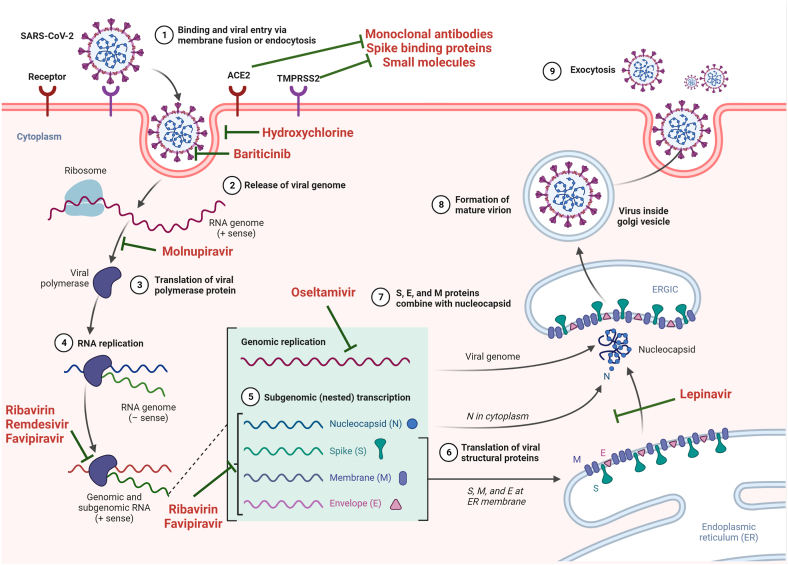
**Entry mechanism of severe acute respiratory syndrome coronavirus-2 (SARS-CoV-2) and target drugs against SARS-CoV-2**: The SARS-CoV-2 enters through major routes; When angiotensin-converting enzyme 2 (ACE2) and transmembrane protease serine 2 (TMPRSS2) co-expressed on the host cell surface, SARS-CoV-2 binds to the ACE2 and get activated by TMPRSS2 via proteolytic cleavage to mediate virus-cell fusion (which can be inhibited by monoclonal antibodies, spike (S) binding protein, and small molecules). **Step 1**-The SARS-CoV-2 enters the host cell receptor via its spike (S) protein. After receptor binding, the SARS-CoV-2 enters the cytosol with the help of the S protein (which can be blocked by Hydroxychloroquine and Baricitinib). Once the viral genome enters the cytoplasm (**Step 2**) (which can be blocked by Molnupiravir), translation of the viral genome (**Steps 3 & 4**) occurs and forms a viral replication-transcription complex (**Step 5**) directly from sub-genomic RNA (+sense) (which can be blocked by Ribavirin, Remdesivir, and Favipiravir). This viral replication-transcription complex contains Envelope (E), S, and Membrane (M) proteins (which can be blocked by Oseltamivir, Ribavirin, and Favipiravir) that will translate from the RNA (**Step 6**) and will enter the endoplasmic reticulum (**Step 6**) and move to the endoplasmic reticulum-golgi intermediate compartment (ERGIC) (which can be blocked by Lepinavir) (Step 7). The viral replication-transcription complex contains nucleocapsid (proteins that interact with M proteins in the ERGIC and form a mature virion (Step 8). This mature virion will move outside the cell via the exocytic pathway (Step 9).

The S protein of SARS-CoV-2 binds to the ACE2 receptor on the surface of host cells, first through the S1 RBD. S1 is then released from the viral surface, allowing S2 to fuse to the host cell membrane. This process needs the S protein to be activated by cleavage at two sites (S1/S2) with the help of the two proteases furin and TMPRSS2. Furin cleaves at the S1/S2 cites and creates conformational changes in the viral S proteins and TMPRSS2 cleavage at S1/S2 cites permit the virus to enter into the host cells. During this process, SARS-CoV-2 enters host cells and contributes to its rapid spread, severe symptoms, and high fatality rates in infected persons [26] (Fig. 2). The mechanism of SARS-CoV-2 entry and viral replication is illustrated in Fig. 3.

## 5 SARS-CoV-2 variants

Adaptive mutations in the virus-related genome can modify the virus's pathogenic potential. Even a single amino acid variation can significantly disturb the virus' aptitude to evade the immune system and complicate the vaccine development against the virus. Viruses like SARS-CoV-2 are prone to hereditary evolution while acclimating to novel human hosts with the effect of one or more mutations, which results in the appearance of multiple novel variants with different characteristics than ancestral strains. As multiple novel variants of SARS-CoV-2 have been found, the center for disease control and prevention (CDC) and WHO have each set up a cataloguing system to distinguish the emerging variants of SARS-CoV-2 into variants of concern (VOCs) and variants of interest (VOIs). Based on recent reports by WHO, five VOCs, such as Alpha (B.1.1.7), Beta (B.1.351), Gamma (P.1), and Delta (B.1.617.2), and Omicron (B.1.1.529) (Table 1) and eight VOIs such as Epsilon (B.1.427 and B.1.429), Eta (B.1.525), Kappa (B.1.617.1), Mu (B.1.621), Lambda (C.37), Theta (P.3), Zeta (P.2), and Lota (B.1.526) (Table 2) were identified, which are summarized in detail below.

**Table 1:** **Details of variants of concerns of SARS-CoV-2**. The details on the name of the variants of concerns, synonyms, with their mutations.

Variants of concerns	Synonyms	Mutations										
		ORF1ab	Spike	ORF3a	ORF3b	E	M	ORF6	ORF7a	ORF7b	ORF8	N
Alpha	B.1.1.7	**Nsp3**: T1001I, A1708D, I2230T **Nsp6**: Del 3675-3677 **RdRp**: P4715L	Del 69-70 Del 144 N501Y A570D D614G P681H T716I S982A D1118H	–	–	–	–	–	–	–	R52I Q27 Y73C	D3L S235F
Beta	B.1.351	**Nsp1**: T265I **Nsp3**: K1655 N **Nsp6**: Del 3675-3677 **3CL**: K3353R	L18F D80A D215G K417 N N501Y D614G A701V	–	–	P71L	–	–	–	–	–	T205I
Gamma	P.1	**Nsp3**: S1188L, K1795Q **Nsp6**: Del 3675-3677 **Nsp13:** E5665D **RdRp**: P4715L	L18F T20 N P26S D138Y K417 N/T E484K N501Y D614G H655Y T1027I V1176F	S253P	–	–	–	–	–	–	E92K	P80R
Delta	B.1.617	**Nsp3**: A1306S, P2046L, P2287S **3CL**: K3255R **Nsp12**: P5715L, G5063S **Nsp13:** P4501L **Nsp14:** A6019V	T19R Del 156 Del 157 R158G L452R T478K D614G P681R D950 N	S26L	–	–	I82L	–	V82A T120I	–	–	D63G R203 M D377Y
Omicron	BA.1	**Nsp3**: K856R, SL2083I, A2710T **3CL**: T3255I, P3395H **Nsp6**: Del 3674–3676, I3758V **RdRp:** P4715L **Nsp14:** I5967V	A67V Del 69–70 T95I GVYY142-145D NL211-212I ins214EPE G339D S371L S373P S375F K417 N N440K G446S S477 N T478K E484A Q493R G496S Q498R N501Y Y505H T547K D614G H655Y N679K P681H N764K D796Y N856K Q954H N969K L981F	–	–	T9I	D3G Q19E A63T	–	–	–	–	P13L Del 31-33 R203K G204R
	BA.2	**Nsp1**: S135R **Nsp3**: T842I, G1307S **Nsp4**: L3027F, T3090I, L3201F **Nsp6**: Del 3675-3677 **3CL**: T3255I, P3395H **RdRp:** P4715L **Nsp13:** R5716C **Nsp14:** I5867V **Nsp15:** T6564I	T19I LPPA24-27S G142D V213G G339D S371F S373P S375F T376A D405 N R408S K417 N N440K S477 N T478K E484A Q493R Q498R N501Y Y505H D614G H655Y N679K P681H N764K D796Y Q954H N969K	T223I	–	T9I	Q19E A63T	D61L	–	–	S84L	P13L Del 31-33 R203K G204R S413R

**Table 2:** **Details of variants of interest of SARS-CoV-2**. The details on the name of the variants of interests, synonyms, with their mutations.

Variants of Interest	Synonyms	Mutations										
		ORF1ab	Spike	ORF3a	ORF3b	E	M	ORF6	ORF7a	ORF7b	ORF8	N
Epsilon	B.1.427	T265I P314L D1183Y	S13I W152C L452R D614G	Q57H	–	–	–	–	–	–	–	T205I
Zeta	P.2	-	E484K D614G V1176F	–	–	–	–	–	–	–	–	–
Eta	P.1	L4715F	A67V Del 69–70 Del 144 E484K D614G Q677H F888L	S253P	–	I82T Del 11288-11289 Del 21765 Del 28278	–	–	–	–	E92K	P80R
Theta	B.1.617	L3201P D3681E L3930F P4715L	E484K N501Y D614G P681H E1092K H1101Y V1176F	–	–	–	–	–	–	–	K2Q	R203K G204R
Iota	B.1.526	Del: 3675-3677	T95I D253G D614G	–	–	–	–	–	–	–	–	–
Kappa	B.1.617	–	G142D E154K L452R E484Q D614G P681R Q1071H	–	–	–	–	–	–	–	–	–
Lambda	C.37	–	G75V T76I Del 246–252 L452Q F490S D614G T859 N	–	–	–	–	–	–	–	–	–

### 5.1 Variant of concerns

#### 5.1.1 Alpha (B.1.1.7)

In late December 2020, a novel SARS-CoV-2 strain (B.1.1.7), also termed the alpha or GRY strain (GR/501.v1), was identified in the UK based on the whole genome sequence (WGS) of samples from patients diagnosed with SARS-CoV-2 [27,28]. The B.1.1.7 variant carries 23 different mutations in the viral genome than the original SARS-CoV-2 strain. Of these, eight mutations, such as N501Y, A570D, P681H, S092A, D1118H, T7161I, Δ69-70 deletion, Δ144 deletion, and S982A have been detected in the spike (S) protein. The N501Y mutation in the B.1.1.7 variant leads to an amino acid conversion from asparagine to tyrosine at position 501 in the ACE2 receptors of the S protein, which boosts the virus to enter the host cells [[29], [30], [31]]. Recently, this N501Y mutation has been described as variant 501YV2 in South Africa. The remaining mutations in the S protein cause the virus to spread rapidly in hosts. A recent study reported that the mortality ratio of patients infected with the B.1.1.7 variant was 2.26 times higher (Hazard ratio (HR); [95% confidence interval (CI): 1.32 to 3.89) than the existing SARS-CoV-2 strain (1.61). Therefore, WHO declared this alpha variant B.1.1.7 as a VOC [[32], [33], [34]].

#### 5.1.2 Beta (B.1.351)

B.1.351 is also called a GH501Y.V2 variant with multiple spike mutations, which was first identified in South Africa in October 2020 [35]. The B.1.351 variant carries 9 different mutations (D80A, D215G, R2461, K417 N, E484K, A701V, L18F, D614G, and L18F) in the S protein, of which 3 mutations, such as K417 N, E484K, and N501Y, are positioned in the receptor-binding domain (RBD) in the S1subunit of the S protein, which boosts the virus to enter the host cells [29,36,37].

#### 5.1.3 Gamma (P.1)

The third VOC is the P.1 variant (Gamma variant or GR/501), first identified in Brazil in December 2020 [38]. This P.1 variant carries 17 different mutations in the viral genome than the original SARS-CoV-2 strain. Of these, the S mutations has 10 mutations including L18F, P26S, R190S, H655Y, T10207I V11176, T20 N, E484K, K417T, D138Y, and N501Y [38]. In those, 3 mutations such as E484K, K417 N, and L184 are positioned in the RBD in ACE receptors like beta (B.1.351) and boost the virus to enter the host cells [38].

#### 5.1.4 Delta variant (B.1.617.2)

The B.1.617.2 variant, also called the Delta variant, was first identified in India in December 2020. The WHO and CDC declared this variant as a fourth VOC because of its highly infectious nature, which is more than 2X as infectious as earlier variants [39]. This B.1.617.2 variant is also responsible for the deadly second wave of COVID-19 infections in many countries, particularly India. The B.1.617.2 variant carries 10 different mutations such as T19R, Δ156-157deletion, L452R, T614G, P781R, R158G, L452R, D950 N, and G142D in the S protein [39].

#### 5.1.5 Omicron variant (B.1.1.529)

The fifth VOC is an Omicron variant (B.1.1.529), first identified in South Africa in November 2021 [40,41]. This Omicron variant has 18,621 mutations. More than 17,703 (97%) of these mutations are in the coding region, while the other 558 (3%) are in the extra-genic region. Because of its different genome construction than the existing variants, the WHO and CDC quickly declared this Omicron variant a VOC. In those, more than 30 mutations (T91, E31del, S33del, R203K, P13L, G204R, Q19E, A63T, Y145del, Y143del, T95I, G142D, H69del, A67V, N501Y, G496S, Y505H, E484A, T478K, S477 N, N440K, K417 N, S371L, S373P, G339D, D796Y, N969K, L981F, Q954H, and S375F) were detected in the S proteins and mostly located in the RBD [42,43]. Recently, investigators identified some worrying mutations, such as N501Y, K417 N, D641G, and T478K, along with the novel mutations in the Omicron variant results in increased infections all over the world.

#### 5.1.6 XE, XD, and XF variant

The Xe variant was first detected in the UK in January 2022. The Xe variant is the recombinant BA.1 BA.2 Omicron variant and harbors 3 mutations: NSP3–C324IT, V1069I, and NSP12–C14599T. The WHO classifies this Xe variant as a VOC.

The XD and XF sub variants share genetic material with the previous Delta AY.4 and Omicron BA.1 variants. The XD sub variant was first detected in Belgium, Denmark, and France. The XF sub variant was first detected in the UK in 2022.

### 5.2 Variant of interests

#### 5.2.1 Epsilon variant (B.1.427 and B.1.429)

Epsilon variants (B.1.427 and B.1.429), also known as CAL.20C, emerged in the US in June 2020 [39]. This epsilon variant has five mutations, such as I4205V and D1183Y in the *ORF1ab* gene and W152C, S131, and L452R in the S protein. In these, the L452 mutation was considered as VOI mutation because it helps the virus to bind to the host cells more effectively [39]. This epsilon variant is now found in the US and 29 other countries. Due to their increased transmissibility, WHO and the CDC considered these strains (B.1.427 and B.1.429) a variant of interest [39].

#### 5.2.2 Zeta variant (P.2)

Zeta variant (P.2), also known as B.1.1.28.2, first emerged in Brazil in April 2020 [44]. The Zeta variant carries key mutations, such as L18F, P26S, E484K, D614G, S929I, T20 N, F157L, and V1176F, in the S protein [45]. The WHO and CDC consider this variant as VOI due to its latent decrease in neutralization by antibody treatments and vaccine sera [45].

#### 5.2.3 Eta variant (B.1.525)

Eta variant (B.1.525) is also termed UK1188, 21D, or 20A/S: 484K and was first detected in New York in November 2020 [46]. This variant harbors S mutations like A67V, Δ69-70 del, Δ144, D614G, Q677H, F888L, T951, A701V, L5F, S477 N, and E484K and does not harbor the N501Y mutation, which originates in the alpha, beta, and gamma variants [46]. But it harbors the same E484K mutation as found in the alpha, beta, and gamma variants, and it also carries the same Δ69-70 del, N439 variant (B.1.141 and b.1.258), and Y453F variant as in the alpha variant [46]. However, the Eta variant differs from previous variants by harboring E483K mutation and a novel F888L mutation (substitution of amino acid phenylalanine (F) with leucine (L) in the S2 domain of the S protein) in the viral genome [46]. This Eta variant has also been considered VOI due to its latent decrease in neutralization by antibody treatments and vaccine sera.

#### 5.2.4 Iota variant (B.1.526)

Iota variant (B.1.526), also called 21F, was first detected in New York in November 2020. This variant harbors the same S mutations (A67V, Δ69-70 del, Δ144, D614G, Q677H, F888L, T951, A701V, L5F, S477 N, E484K) as the Eta variant [39]. This Iota variant has also been considered VOI because of its latent decrease in neutralization by antibody treatments and vaccine sera.

#### 5.2.5 Theta variant (P.3)

Theta variant (P.3) is also called GR/102K.V1, which was first detected in the Philippines and then in Japan on February 2021. This variant carries 3 spike mutations, Δ141-143del, N501Y, and P681H, and is classified as the VOI by the CDC and WHO due to its lower threat than other strains [47].

#### 5.2.6 Kappa variant (B.1.617.1)

Kappa variant B.1.617.1 was first detected in India in October 2020. This variant harbors T951, E154K, L452, E484Q, D614G, G142D, and Q1071H mutations in the viral genome. This kappa variant has three notable substitutions, such as L452R (leucine to arginine substitution at position 452) [39,48], E484Q (glutamic acid to glutamine substitution at position 484) [47,49], and P681R (proline to alanine at position 681) in the S protein [47]. However, it poses only a lower threat to the world, so CDC and WHO consider this strain a VOI. The symptoms of the Kappa variant are fever, cough, rashes, sore eyes, and a runny nose.

#### 5.2.7 Lambda variant (C.37)

Lambda variant C.37 appeared first in South America in June 2020. This variant harbors L454Q, 7 amino acid deletion (arginine 246 to guanine 252) mutations in the S protein, and 3 amino acid deletions in the ORF1ab at positions 3675–3677 [39,48]. This Lambda variant poses only a limited threat; therefore, the CDC and WHO consider this strain a VOI.

#### 5.2.8 Mu variant (B.1.621)

Mu variant (B.1.621) was first detected in South Africa in August 2021. This variant harbor mutations like ST95I and E484K, as seen in the VOI and VOC. It has one amino acid substitution (Y144T and Y145S) at position 146 in the S protein and four base-frameshift mutations in the *ORF3* gene (G26158 to A26161) [39,48]. Due to its lower threat, the CDC and WHO declared this Mu variant (B.1.621) as the VOI.

## 6 Antiviral drugs for SARS-CoV-2

In the early stages of the SARS-CoV-2 outbreak, there were no effective vaccines or antiviral drugs to reduce the risk of COVID-19 progression. Therefore, the field expert proposed some existing antiviral drugs (used during MERS and SARS-CoV-1 infections) and developed some effective drugs to control SARS-CoV-2 (Fig. 3 and Table 3), which are summarized in detail below.

**Table 3:** Details of NIH-recommended antiviral therapeutics drug for non-hospitalized patients with mild-moderate COVID-19 (including Omicron variant) at high risk of progression to severe disease. The details on the name of the drug, mode of action, doses, route with the start of symptom onset, age, and efficacy.

S. No.	Name of the drug	Mode of action	Doses	Route	Symptom onset	Age	Efficacy
1	Paxlovid (Ritonavir-Boosted Nirmatrelvir)	n = protease inhibitor, halts viral replication of all known coronaviruses r = CYP 3A4 inhibitor (boosting agent)	300 mg	Orally	Start after 5th day	Age above 12 years	88%
2	Remdesivir	Prodrug of adenosine analog ends viral RNA transcription	200 mg (day 1) 100 mg (2nd and 3rd days)	Orally	Start after 7th day	Age above 12 years	87%
3	Bebtelovimab	Monoclonal antibody	175 mg	IV infusion	Start after 10th day	Age above 12 years	–
4	Molnupiravir	The prodrug of ß-D-N4hydroxycytidine (NHC) induces lethal RNA viral mutagenesis	800 mg	Orally	Start after 5th day	Age above 18 years	30%
5	Sotrovimab	Monoclonal antibody	500 mg	IV infusion	Start after 10th day	Age above 12 years	85%

### 6.1 Paxlovid (PF-07321332)

Paxlovid is an antiviral drug discovered by Pfizer, combining nirmatrelvir (SARS-CoV-2 3C-protease inhibitor that prevents the growth of the virus) and ritonavir (a boosting agent, which helps nirmatrelvir work better) [50]. Early tests of Paxlovid against SARS-CoV-1 showed good results, so Pfizer tested it on SARS-CoV-2 to check its efficacy. Laboratory tests showed promising preliminary results, lowering the risk of hospitalization and death in unvaccinated people by 88%. Researchers also observed that Paxlovid is very effective against Omicron. In the second trial (June 2022), they selected vaccinated people who had significant risk factors for COVID-19. But the results were disappointing for Pfizer, which does not show any efficacy in speeding up recovery from COVID-19 infections. However, Pfizer applied for full authorization from the Food and Drug Administration (FDA) on June 30, 2022, using these two preliminary study results [50]. In August 2022, an Israeli scientist analyzed the efficiency of Paxlovid. In brief, they selected 3902 patients and provided Paxlovid. In 65 patients (age above 65), Paxlovid reduced hospitalization by 73%, but in patients aged 40–64, Paxlovid did not reduce the viral infection [50]. From these results, the National Institutes of Health (NIH) and WHO strongly recommend Paxlovid drug for older people with a high risk of hospitalization. Therefore, FDA approved the Paxlovid drug in December 2021 for older (age above 65) SARS-CoV-2 infected patients.

### 6.2 Lopinavir/ritonavir

Lopinavir/ritonavir is a human immunodeficiency virus (HIV) protease inhibitor that is being used for HIV viral infection. This lopinavir hinders the action of 3CL protease in HIV through the C2-symmetric pocket [51]. Hence, China investigated the efficiency of the lopinavir/ritonavir drug against SARS-CoV-2 infection [9]. They selected 199 COVID-19-infected patients (oxygen saturation <94% in ambient air) and gave 400/100 mg of lopinavir/ritonavir for 28 days [9,52]. After 28 days of clinical trials, they could not find any differences in the lopinavir/ritonavir-received SARS-CoV-2 infected patients [9,52]. These data clearly show that lopinavir/ritonavir cannot be used to reduce the viral titre of the SARS-CoV-2 infected patients.

### 6.3 Chloroquine/hydroxychloroquine

Chloroquine/hydroxychloroquine is a disease-modifying anti-rheumatic drug (DMARD) that has been utilized to treat malaria caused by *Anopheles* mosquitoes [53,54]. It also treats rheumatoid arthritis, discoid lupus erythematosus, and Porphyria cutanea tarda diseases. Therefore, chloroquine/hydroxychloroquine has been studied for treating SARS-CoV-2 infection. In 2020, Wang et al. reported that chloroquine/hydroxychloroquine effectively controls SARS-CoV-2 infection in host cells [53]. Following this, Gautret et al., 2020 conducted an open-label, non-randomized trial using 36 SARS-CoV-2-infected patients in France. They provide 200 mg of chloroquine/hydroxychloroquine thrice a day for 10 days [55]. After 10 days, the viral load was considerably less in treated patients than in untreated patients [55]. In addition, they treated SARS-CoV-2 infected patients by providing 500 mg of chloroquine/hydroxychloroquine and observed 100% viral clearance on the 6th day [55]. In another study, 400 mg of chloroquine/hydroxychloroquine (twice on day 1, followed by 200 mg twice daily for 5 days) was provided orally to SARS-CoV-2 infected patients in New York [56]. Unfortunately, people treated with Chloroquine/hydroxychloroquine faced severe prolonged QTc, which can cause sudden cardiac arrest and arrhythmia [56]. Therefore, experts informed that chloroquine/hydroxychloroquine should be used only with proper precaution.

### 6.4 Remdesivir

Remdesivir is an RNA polymerase inhibitor that binds to a virus-related RNA-dependent RNA polymerase and prevents RNA virus replication by ending RNA transcription [57]. Therefore, remdesivir was used against the SARS-CoV-2 infection [58]. Initially, remdesivir was tested in SARS-CoV-2 infected model rhesus macaques. The remdesivir-treated rhesus macaque had a lower viral level in the lungs than the untreated rhesus macaque. Hence, the CDC and WHO considered remdesivir a potential drug to treat SARS-CoV-2 infection at the beginning of the epidemic. Holshue et al. (2020) initially tested the efficiency of remdesivir in the US, but the result was unclear [59]. Then, Grein et al. (2020) examined the efficiency of remdesivir at Australia with 53 patients that exhibited 68% improvement in oxygen care for COVID-19 patients, 47% were discharged, and 13% of patients died [60]. Following the above result, China conducted a trial using remdesivir against SARS-CoV-2 infections. Still, they could not find any evidence about the efficacy of remdesivir (reducing the recovery time and deaths) [61]. After that, Beigel et al. (2020) groups from Italy conducted a trial using remdesivir against SARS-CoV-2-infections and found effective results. This result revealed that remdesivir reduced the recovery time from 15 days to 11 days in hospitalized SARS-CoV-2 infected patients [62]. Following these results, the National Institute of Allergy and Infectious Diseases (NIAID) from the US declared remdesivir a better drug for treating hospitalized SARS-CoV-2 infected patients. Recent studies also reported that remdesivir reduces the likelihood of hospitalization by 87%. In brief, 562 unvaccinated volunteers (above 60) were selected for this study. Each volunteer received 100 mg of remdesivir for three days. After three days, remdesivir lowers the risk of hospitalization and death by 87%. This shows that remdesivir could reduce the infection of the SARS-CoV-2 virus, including Omicron. By analysing these results, the FDA approved remdesivir for non-hospitalized children, adults, and older people on April 25, 2022. Recently, researchers also boost the remdesivir by adding baricitinib to treat the COVID-19 infection effectively. However, the first trial phase is currently in progress with the combination of remdesivir/baricitinib, which might combat SARS-CoV-2 and its variants efficiently in the future.

### 6.5 Favipiravir (Avigan)

Favipiravir is a synthetic purine nucleotide prodrug that inhibits RNA polymerase and is used against the influenza virus [63]. Wang et al. (2020) verified the activity of favipiravir against SARS-CoV-2 with a half-maximal effective concentration (EC_50_) of 61.88 μM/L [53]. Next, various dosages have been provided based on the infection symptoms (2400–3000 mg and 1200-1,800 mg for 12 h) [63]. This favipiravir drug reduces the viral load in SARS-CoV-2-infected patients more than the other antiviral drugs [63]. In February 2020, China investigated the use of favipiravir for the treatment of SARS-CoV-2 infection by providing 1600 mg for the first day (every 12 h), followed by 600 mg for 10 days (every 12 h), and observed a significant difference between hosts with mild fever and cold [64]. However, only a few clinical trials of favipiravir to treat SARS-CoV-2 infection have been conducted, necessitating additional research.

### 6.6 Molnupiravir (Lagevrio)

The antiviral medication molnupiravir, sometimes referred to as Lagevrio, was developed for COVID-19 by Ridgeback Biotherapeutics and Merck. On December 23, 2021, the United States authorized its use for emergency purposes [65]. The NIH guidelines only suggest using molnupiravir if Paxlovid or Remdesivir is unavailable because it is less effective than other therapies for COVID-19.

Molnupiravir was initially created to combat the flu. Ridgeback Biotherapeutics and Merck checked the efficacy of molnupiravir against SARS-CoV-2 [66]. They performed two phases of trial that showed promising results by reducing the risk of hospitalization and death by half [66]. Therefore, in November 2022, the United Kingdom approved Molnupiravir as an emergency drug. Then, in March 2022, WHO also approved to use of Molnupiravir drug in high-risk patients. India also did a second trial of Molnupiravir. The results were positive, reducing the risk of hospitalization by 65%. India also informed that the Molnupiravir drug is effective against the Omicron variant. However, the Phase 3 trials are still ongoing [66].

### 6.7 Xocova (S-217622)

The Japanese company Shionogi has developed an antiviral drug called Xocova (S-217622). Shionogi applied for authorization to use Xocova against SARS-CoV-2 [67]. But the Japanese regulators announced in July 2022 that they would delay the approval of Xocova until the results of the Phase 3 trial in known [68]. Therefore, on March 31, 2022, Shinogi started a global Phase 3 trial. If the trial results are promising, it may be added to the list of SARS-CoV-2 drugs [68].

### 6.8 Ensovibep

Ensovibep is a recent antiviral drug developed by a Swiss company. This drug was developed to target the S protein of SARS-CoV-2, which can be delivered in a single injection. In the phase 2 trial (the result of the phase 1 trial was not revealed), they chose 407 volunteers and found that the Ensovibep was effective, which cut the risk of hospitalization by 78%. The phase 3 trial is in progress, requiring additional studies against the Omicron variant.

### 6.9 Baricitinib

In April 2021, European Medicine Agency (EMA) suggested using baricitinib to treat hospitalized COVID-19 patients who require additional oxygen. This medicine is currently being used to treat moderate to severely active rheumatoid arthritis and atopic dermatitis (AD) in elderly patients. Janus kinases (JAK1 and JAK2) are selectively and permanently inhibited by this drug, baricitinib [69,70]. JAKs function as part of the intracellular JAK-signal transducer and activator of the transcription (JAK-STAT) signaling pathway. They phosphorylate and activate STATs, stimulating the expression of genes encoding proteins involved in inflammation and hematopoiesis. JAK inhibition may diminish an undesirable inflammatory response. Therefore, researchers checked the efficacy and safety of baricitinib against hospitalized COVID-19 patients. Baricitinib was given orally at a dose of 4 mg daily for 14 days until discharged from the hospital [71]. Baricitinib showed promising results by decreasing the mortality rate than the control group. In brief, a total of 357 patients underwent randomization (with 149 assigned to Baricitinib and 216 to placebo). Those who received Baricitinib for 28 and 68 days had a lower mortality of 8% (n = 62) and 10% (n = 79) than the placebo patients mortality rate 13% (28 days) and 15% (68 days) [71]. However, each group had severe infections and other side effects. In another study, researchers found that the combination of baricitinib with hydroxychloroquine and lopinavir results in decreased mortality and increased recovery rate than the hydroxychloroquine and lopinavir without baricitinib [72]. The recommendation for using the above-mentioned antiviral drugs according to disease severity is summarized in Table 1.

### 6.10 Monoclonal antibodies against S protein of SARS-CoV-2

Monoclonal antibodies (MAbs), which target the SARS-CoV-2 are a potential antibody for COVID-19. As an emergency outbreak of COVID-19, FDA approved three antibodies such as bamlanivimab, imdevimab, and casirivimab for treating SARS-CoV-2-infected patients (mild to moderate (aged above 12). These antibodies bind directly to the S protein and block the S protein's ability to bind to the host receptor (ACE2) and inhibit the virus' ability to infect host cells.

#### 6.10.1 Bamlanivimab (LY3819253) and etesevimab (ly3832479)

In neutralizing antibodies trial, researchers found that bamlanivimab combined with etesevimab decreased viral load, hospitalizations, and deaths than the Bamlanivimab [73,74] Therefore, FDA in September 2021, approved to provide 700 mg of bamlanivimab and 1400 mg of etesevimab as a single dose to adults and pediatric COVID-19 infected patients (aged above 12 years) [73,74]. However, some cases of COVID-19 symptoms were observed after bamlanivimab administration, involving signs and symptoms such as fever, hypoxia, breathing difficulties, arrhythmia, and fatigue. Some patients deserved to be hospitalized. It is still unknown if these symptoms were caused by bamlanivimab or due to COVID-19 infections [73,74].

#### 6.10.2 Casirivimab (REGN10933) and Imdevimab (REGN10987)

Casirivimab and Imdevimab are human monoclonal antibodies that bind to the RBD of the S protein and reduced the viral replication [75]. Researchers found that combination of casirivimab and imdevimab blocks the RBD and minimizes the virus replication. These two combinations were named as REGN-CoV2 [76]. Scientists evaluated the efficacy of REGN-CoV2 using 799 adult COVID-19 patients in November 2021 [77]. The recommend REGN-CoV2 dosage is 600–1200 mg for mild to moderate COVID-19 patients for adolescents (aged above 12) and adults, who does not require additional oxygen. They selected 799 adult COVID-19 patients for this evaluation. The patients treated with REGN-CoV2 had lower viral load than the placebo group after seven days [78]. Through these results, WHO and EMA recommended REGN-CoV2 for mild to moderate COVID-19 patients.

#### 6.10.3 Regdanyimab (Regkirona)

Regdanyimab is a recombinant human monoclonal antibody, which binds to the RBD of the S protein [79,80]. The ongoing trial of Regdanyimab lowers the viral load and speeds up the recovery time of COVID-19-infected patients. Regdanyimab received patients 3.1% (14 out of 446) were hospitalized and died within 28 days of treatment compared to 11.1% (48 out of 434) of placebo patients. Regdanyimab is provided as a single dose of 40 mg/kg for seven days. The commonly reported side effects are headache, rash, fever, hepatitis, etc. Regdanyimab did not work efficiently for ICU persons. In November 2021, Regdanyimab was approved by EMA for treating adult patients who do not require supplemental oxygen. Many other antiviral drugs and antibodies are still in process to treat COVID-19 infected patients [79,80].

Details of NIH-approved antiviral therapeutics drug (mode of action, doses, doses, route with the start of symptom onset, age, and efficacy) for non-hospitalized patients with mild-moderate COVID-19 (including Omicron variant) are described in detail in Table 3.

## 7 Vaccine

A vaccine is an organic substance that shelters humans from infections caused by harmful viruses or bacteria. Developing novel vaccines against these harmful pathogens (viruses or bacteria) takes nearly 10 to 15 years. Hence, developing a vaccine against the current SARS-CoV-2 strain becomes a crucial challenge for researchers. However, scientists from various countries came up with emergency vaccines against SARS-CoV-2 and received emergency use authorization (EUA) approval, which is summarized in detail below.

### 7.1 Stages of vaccine

The development of novel vaccines undergoes numerous screening and evaluation stages. The preliminary stage of the discovered vaccine will be tested only on animals to evaluate its safety and potential to inhibit infections. If the developed vaccine triggers an immune response in animals, then the vaccine will be tested in humans in three phases.

#### 7.1.1 Phase 1

Phase 1 is also called the “safety stage” (first stage), where the vaccines will be injected into a small number of healthy volunteers. This phase 1 confirms vaccine safety, determines the right dosage, and confirms whether the vaccine generates an immune response in humans [81].

#### 7.1.2 Phase 2

Phase 2 is also called the “expanded safety stage” (second stage). During Phase 2, the developed vaccine will be injected into 100 volunteers (same age and sex). Phase 2 confirms the ability of the vaccine's safety and the vaccine-generated immune response in humans. In addition, Phase 2 will confirm the right dosage of vaccines that should be injected into humans [81].

#### 7.1.3 Phase 3

Phase 3 is also termed the “efficacy stage” (third stage), where the vaccine will be injected into 1000 healthy peoples. Phase 3 stage confirms the ability of the vaccine's safety and the vaccine-generated immune response in humans with dosage. Once this Phase 3 has been screened and evaluated safely, the vaccine will be reviewed and approved by the FDA or EUA. Following this process (nearly 1–2 years), the vaccine will be manufactured and used for the public [81].

### 7.2 DNA-based vaccines

**Fig. 4:**
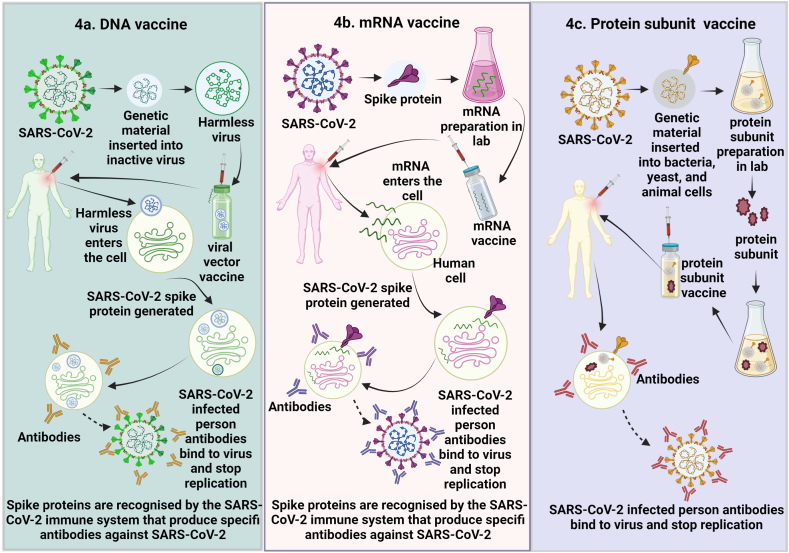
**Different types of vaccines used for severe acute respiratory syndrome coronavirus-2 (SARS-CoV-2). A) mRNA vaccine** works by using a piece of mRNA that corresponds to a viral protein. After receiving the mRNA vaccination, human cells will produce a foreign viral protein. As part of the normal immune system, the immune system of the body will be able to recognize the foreign viral protein and will start to produce antibodies against the foreign viral protein. These antibodies will protect humans against SARS-CoV-2 infection. **B) In the DNA vaccine**, genetic material from the SARS-CoV-2 is placed in an altered form (viral vector). When this viral vector is injected into cells, it will deliver the genetic material of SARS-CoV-2 and will start to produce a foreign viral protein. The normal immune system of the body will recognize the foreign viral protein and will start produce antibodies against the foreign viral protein. These antibodies will protect humans against SARS-CoV-2 infection. **C) Protein subunit vaccine. T**his vaccine includes only slices of a virus, which stimulates the immune response. This vaccine contains harmless S protein and once our body recognizes the S proteins, it will create antibodies and defensive white blood cells. If humans infected with SARS-CoV-2, these antibodies would fight against the SARS-CoV-2.

Numerous DNA vaccines have been developed against existing cytomegalovirus (CMV), Venezuelan equine encephalitis virus (VEEV), Zika virus (ZIKV), Ebola virus (EBOV), MERS-CoV, influenza virus, and human immune viruses. As DNA vaccines showed promising control strategies for existing viruses, researchers evaluated the immunogenicity of DNA vaccines against SARS-CoV-2 (Fig. 4b).

#### 7.2.1 GX-19 and GX-19 N

GX-19 and GX-19 N are SARS-CoV-2 DNA vaccines developed by the Genexine consortium. The GX19 vaccine contains only a plasmid encoding the SARS-CoV-2 “S” protein, whereas the G19 N vaccine contains plasmid encoding the SARS-CoV-2 “S”, “RBD” and ”N″ proteins [82]. In the Phase 1 trial, 40 volunteers received 1.5 and 3 mg doses of the GX19 (NCTO44445389) vaccine via intramuscular injection (IM). The G19 vaccine showed a low viral titer of neutralizing antibodies (NAb) in the treated persons [82]. In GX19 N (NCTO4715997) vaccine-injected persons (21 volunteers), NAb increased significantly more than the G19 vaccine. However, GX19 and GX19 N vaccines considerably enhanced T-cell responses [82]. The GX19 vaccine enhanced 50% of S-specific T-cell responses in the clinical trial, and the GX19 N vaccine enhanced 75% of S- and N-specific T-cell responses and induced stronger T-cell immune responses in the clinical trial [82].

#### 7.2.2 INOVIO's (IN-4800)

INOVIO's (IN-4800) is also a DNA vaccine developed by Inovio Pharmaceuticals (America) against SARS-CoV-2. Phase 1 trial of the INOVIO-DNA vaccine was tested with 40 healthy persons. The INOVIO-DNA vaccine was injected using intradermal (ID) injections followed by electroporation to confirm cell uptake [83]. The INOVIO-vaccinated patients exhibited higher immune responses (34 out of 36 patients (94%) [83,84]. However, the complete clinical trials of the INOVIO-DNA vaccine are still unclear.

#### 7.2.3 Zydus Cadila (ZyCoV-D)

Zydus Cadila (ZyCoV-D) is another DNA vaccine that contains a plasmid encoding the SARS-CoV-2 “S” protein. In the Phase 1 clinical trial, 126 volunteers were selected, and three doses (1 or 2 mg) of ZyCoV-D were injected through ID injections. The ZyCoV-D was safe in the 1st clinical trial, but the NAb was very low (<40). Based on the enzyme-linked absorbent spot (ELISPOT) assay, the ZyCoV-D vaccine induced higher cellular immune responses at 2 mg. However, it does not induce changes in the IL-2, IL-4, IL-6, IL-10, IFN-γ, TNF-α, and Th-17a cytokine levels. During the phase III clinical trial, the ZyCoV-D vaccine showed 67% protection against symptomatic SARS-CoV-2. India has approved this ZyCoV-D DNA-based vaccine for emergency use against SARS-CoV-2. ZyCoV-D vaccine is the world's first approved DNA vaccine used in humans [85]. There are 11 other DNA vaccines in clinical trials against SARS-CoV-2. In the Phase 3 clinical trials, there were a few minor side effects after the first dose of ZyCoV-D, but the following doses were successful [85].

### 7.3 mRNA-based vaccine

The mRNA vaccine requires only a piece of mRNA that links to a viral protein (a small piece of protein from the virus's outer membrane). Utilizing this piece of mRNA, cells produce viral proteins. As part of a normal immune response, the immune system recognizes that the viral protein is foreign and develops particular proteins called antibodies. These antibodies easily identify viruses and protect our bodies from their virulence. Using this mRNA blueprint, various companies like Moderna and BioTNech/Pfizer formulated mRNA vaccines against SARS-CoV-2 (Fig. 4a).

#### 7.3.1 Moderna mRNA (mRNA 1273)

Moderna is an American company (Cambridge) that developed the mRNA vaccine against SARS-CoV-2 [86]. This Moderna vaccine codes for the “S” protein and destroys the viral particle of SARS-CoV-2 in the vaccinated body [86]. Moderna mRNA was first tested in mice by vaccinating them with 0.01, 0.1, and 1 μg doses [87]. They observed a very high pseudo-virus NAb response in 1 μg doses and a high pseudo-virus NAb response in the S (D614G) protein [87]. Further, it showed a high cytotoxic T cell response along with the hormonal messengers Th1/Th2, which is important because Th2 is linked with vaccine-associated enhanced respiratory disease (VAERD). Due to its high efficacy, Moderna started testing the mRNA vaccine on people [87]. In Phase 1 trials, they selected 45 healthy persons, split them into 3 groups, and supplied 3 different doses (25, 100, 250 μg). One volunteer from group 25 μg and another from group 250 μg tested negative and neglected their second dose [88]. Moreover, the remaining volunteers from each group tested negative only after receiving the double dose [88].

For the Phase 2 trial, they selected 600 healthy volunteers (age 18 and above) and checked the safety and immunogenicity of the vaccine [88]. They split the volunteers into 8 groups based on age and doses of 50 and 100 μg (4 group volunteers were taking 50 and 100 μg of the vaccine, and the remaining 4 group volunteers were taking 50 and 100 μg of the saline-placebo) [88]. After the second dose of vaccination with mRNA-1273, antibody titers are higher than those reported by Pfizer. Neutralizing activity is lower against Delta [54] and Omicron [87] variants than against strains already in circulation [89]. Similar results were also observed in a recent study, which involved the comparison of induction of humoral and cellular responses following the administration of several vaccines [90]. The phase 3 trial was done with 30,000 healthy volunteers (age 18), and they found a lower viral titer in the vaccinated volunteers than in the unvaccinated volunteers [91]. The Moderna mRNA vaccine showed an immune response in volunteers. Following the first dosage of the vaccine, 84% of patients exhibited immediate reactions at the injection site, according to the results of phase 3 clinical trial, whereas 0.8% of patients experienced delayed reactions [92]. In addition, there were other signs like fatigue, chills, headache, nausea, sweating, and muscle spasms [92].

#### 7.3.2 Pfizer-BioNTECH (BNT162b2)

Pfizer is an American company that joined BioNTECH (a German company) (BNT162b2) and developed a novel mRNA vaccine against SARS-CoV-2, which encodes the RBD domain [93]. This vaccine contains the T4 fibrin-derived trimerization domain, enhancing the immune response. In Phase 1 and Phase 2 trials, they selected 45 healthy volunteers (aged 18–55) and split the volunteers into 12 groups for 10, 30, and 100 μg doses of the vaccine [93]. They checked the safety and immunogenicity of the vaccine. The vaccinated volunteers exhibited increased immunoglobulin G (IgG) antibody levels after receiving a second dose [93]. In the volunteers who received 100-μg doses, the IgG level was very high 21 days after receiving the first dose. Further, they could not identify any significant difference in immune response between the 30 and 100 μg doses received by volunteers. This result confirmed the efficacy of the Pfizer vaccine [93].

Moderna and Pfizer vaccines are the first approved mRNA-based vaccines. These two vaccines had an efficacy of greater than 90% up to 5 months after receiving the 2nd dose. In contrast, the other mRNA vaccine, the CureVac vaccine, exhibited only 48% efficacy against SARS-CoV-2 and its variants. After the second vaccination dose compared to the first, the side effects are relatively more frequent such as fatigue, muscular pain, headache, etc.

### 7.4 Non-replicating viral vector vaccines

Viral vector vaccines are developed based on recombinant viral vectors. These recombinant vector-based vaccines induce T cells' strong cellular immune response and increase antibody production in the B cells. Hence, scientists developed non-replicating viral vector vaccines against SARS-CoV-2, which are summarized in detail below.

#### 7.4.1 AstraZeneca (AZD1222/ChAdOx1 nCoV-19)

The University of Oxford and the British pharmaceutical company AstraZeneca joined and developed an AstraZeneca viral vector vaccine against SARS-CoV-2. This non-replicating viral vaccine was called ChAdOx1 and is now called AZD1222 [94]. They conducted Phase 1 and Phase 2 trials on 1077 healthy volunteers (aged 18 to 55). The volunteers received both the AZD1222 vaccine (n = 543) and the placebo meningococcal vaccine MenACWY (n = 534) at a dose of 5 × 1010 viral particles [94]. The dose for this vaccine was provided based on previous experience with MERS infection. The vaccinated volunteers exhibited increased immunoglobulin G (IgG) antibody levels after receiving the second dose [94]. The Phase 3 trial was done with 30, 000 healthy volunteers (age 18), and they found a lower viral titer in the vaccinated volunteers than in the unvaccinated ones. In some people who have received vaccinations, the Delta and Omicron variants elude immunological responses [95,96]. Breathing issues, chest and stomach pain, thrombocytopenia, and myocarditis are some of the side effects of AZD1222 [97].

#### 7.4.2 CanSino's (Ad5-nCoV)

CanSino's Ad5-nCoV is a non-replicating viral vector vaccine that uses the Ad5 adenovirus. This vaccine was successfully used against the Ebola virus. For CanSino's Phase 1 vaccine safety trials against SARS-CoV-2, they selected 108 healthy volunteers (aged above 18) and split them equally into 3 dose groups [98]. The first group received a dose of 5 × 1010 viral particles, the second group received 1 × 1011 viral particles, and the third group received 1.5 × 1011 viral particles [98]. The vaccinated volunteers exhibited increased NAb titer levels after 14 days of vaccination. T cells also induce a strong cellular immune response and increase antibody production in B cells [99]. After these successful results, they conducted Phase 2 vaccine safety trials on 508 healthy volunteers [99]. The volunteers were injected using the IM route with 1 × 1011 viral particles (n = 253), 5 × 1010 viral particles (n = 128), and 5 × 1010 placebo (n = 129) and found increased NAb titer levels, high cellular immune response in T cells in 1 × 1011 viral particles received volunteers [99,100]. In a recent Phase 4 trial, participants who had received two doses of CoronaVac responded better neutralizing antibody titers to the heterologous booster dosage of Ad5-nCov or Convidecia than the homologous booster doses of CoronaVac [101]. The most frequent adverse effects of Ad5-nCoV are fever, exhaustion, headaches, and muscle soreness [98].

#### 7.4.3 Johnson and Johnson (Ad26.CoV2.S)

Johnson and Johnson's (Ad26.CoV2.S) is another non-replication viral vector vaccine, which contains a recombinant, replication-incomplete AD26 vector that encodes the variant of the SARS-CoV-2 “S” protein [102]. Phase 1 and Phase 2 trials were conducted with 2000 healthy volunteers (aged 18 to 59), and they found increased NAb titer levels. The vaccine also induced strong cellular immune responses in T cells during Phases 1 and 2 [102]. They conducted Phase 3 with 44,325 volunteers and found increased NAb titer levels and a high cellular immune response in T cells [102].

The viral vector vaccines such as AZD1222, Ad5-nCoV, and recently invented Sputnik V showed an efficacy of 65–96% against the origin SARS-CoV-2 strain. The AZD1222 vaccine showed more than 89% efficacy against the Alpha, 95% against Beta/Gamma, and 96% against Delta strains. The Ad5-nCoV vaccine showed 60–85% and 64.7% efficacy against the Delta and Beta strains, respectively. The Ad26. CoV2 viral vector vaccines showed only low efficacy of 10.4% against the Beta strain. Overall, the effectiveness of the vaccines reduced the infection of the Delta variant, but it did not reduce the infection of the Omicron strain [90]. The adverse effects of Ad26.CoV2.S includes myocarditis, thrombocytopenia, chest and stomach pain, and difficulty breathing.

#### 7.4.4 Sputnik V (Gam-COVID-Vac)

Sputnik is an adenovirus viral vector vaccine developed for COVID-19 by Gamaleya Research Institute. This vaccine was available all over world [103]. A recent interim analysis of a Phase 3 trial found that the second dose of Sputnik V was 91.6% effective at preventing COVID-19. Recently, Sputnik V was administered intranasally to nonhuman monkeys, and produced a strong immune response [103]. Sputnik's side effects are pain at the injection site, exhaustion, headaches, body aches, fever, sleepiness, and chills [104].

### 7.5 Inactivated vaccine

Inactivated vaccines are another form of vaccine which utilizes the killed viruses that cause disease. The inactivated vaccines typically do not deliver immunity protection like other live vaccines (e.g., vaccines used against small pox, chicken pox, and mumps). Hence, the person needs several doses of inactivated vaccine booster shots to raise immunity against the disease. Scientists developed some inactivated vaccines as an emergency approach to the battle against SARS-CoV-2, which are discussed in detail below.

#### 7.5.1 Sinopharm (BBIBP)

Sinopharm is an inactivated vaccine developed by the Wuhan Institute of Biological Products (WIBP) and the Beijing Institute of Biological Products (BIBP) against SARS-CoV-2. In the Phase 1 clinical trial, 96 volunteers (aged 18 and 59) were selected, and three doses (2.5 μg, 5 μg, and 10 μg) of Sinopharm were injected through ID injections (0, 28, and 56 days) [105]. On day 7, the low dosage received group had adverse reactions of 20.8% (5 out of 24), the medium dosage received group had adverse reactions of 16.7% (4 out of 24), and the high dosage received group had adverse reactions of 12.5% (6 out of 24) [105]. After receiving a third-booster vaccine at day 70, the low, medium, and high dosage groups had a high NAb response. A high level of seroconversion (the development of specific antibodies in the blood serum against infection) was also observed in the volunteers [105].

The phase 2 trial was done with the help of 224 volunteers (aged 18 and 59) [105]. In the Phase 2 trial, they provided vaccines in two dual doses with two different day intervals (days 0 & 14 and days 0 & 21). For days 0 and 14, days 0 and 21 schedules, 5 μg of dosage was injected into 84 volunteers and 28 volunteers in the placebo group (for safety analysis) [105]. The volunteers who received dosage in 0 and 14-day schedules had adverse reactions of 6% (5 out of 84 volunteers group) and 14% (4 out of 28 volunteers group) [105]. The volunteers who received dosage in 0 and 28-day schedules had adverse reactions of 19% (16 out of 84 volunteers group) and 17.9% (5 out of 28 volunteers group) [105]. The volunteers who received dosages on both schedules had high NAb responses. The WIBP and BIBPP also conducted a randomized double-blinded trial with 1120 healthy volunteers and observed high antibody responses [106]. In a phase 3 trial, vaccination effectiveness was estimated to be 73%–78% compared to a placebo. Another study in Thailand evaluated the effectiveness of AZD122, BNT162b2, and mRNA-1273 vaccines (as a booster dose) with BBIBP. After receiving two doses of BBIBP along with a booster dose, it developed neutralizing antibodies that reduced the Delta and Omicron variants by 90% and 70%, respectively [107]. These antibodies also increased the CD4^+^ T cells' ability to produce IFN, increasing the inhibitory effect on the virus [107]. After the first dosage, the BBIBP vaccination frequently causes headaches, lethargy, and soreness at the injection site. During the second dose, it causes lethargy, exhaustion, and tenderness [108].

#### 7.5.2 Covaxin (BBV152)

Covaxin is a vaccine made by the Indian Council of Medical Research and Bharat Biotech to stop the spread of SARS-CoV-2. It stops a virus from spreading. It includes a toll-like receptor agonist adjuvant as well as aluminum hydroxide. There are two intramuscular injections given, separated by 29 days. During a randomized trial, Covaxin showed a 78% success rate against COVID-19 symptoms compared to the placebo group. After six months post-vaccination, 85% of people had antigen-specific CD4^+^ T cells, 50% had CD8^+^ T cells, and these cells also produced a wide range of cytokines [109].

#### 7.5.3 CoronaVac (SinoVac)

SinoVac, or CoronaVac, is also an inactivated aluminum adjuvant vaccine. The Phase 1 clinical trial of SinoVac was randomized and double-blinded with 143 volunteers (aged 18 and 59) [110]. The Phase 2 clinical trial was randomized and double-blinded with 600 healthy volunteers [110]. The selected volunteers were split into two dual-dose schedules (days 0 & 14 and days 0 and 28). The 1st group of volunteers (120) received a 3-μg dose, and the 2nd group (120) received a 6-μg dose. Both doses received by volunteers in two schedules faced severe pain and swelling in the body. Some volunteers also had severe pain at the vaccine-injected spot [110]. However, the pain is gone within 3 days of the vaccination. The NAb responses were also very high in both groups. After receiving the second dose, the 0 and 14 days of scheduled vaccine-injected volunteers had stable Nab levels, but the 0 and 28 days of vaccine-injected volunteers had high NAb levels [110]. After these successful results, the Phase 3 trial was done in China, Pakistan, Brazil, Hong Kong, Indonesia, and Turkey and has been approved in 54 countries worldwide. The results of the Phase 3 trials of SinoVac showed that they were effective against SARS-CoV-2 in terms of IgG response and NAb titer levels [111].

### 7.6 Protein subunit vaccine

This vaccine includes only slices of a recombinant virus, which stimulates the immune response [112]. This vaccine contains harmless S proteins, and once our body recognizes them, it will create antibodies and defensive white blood cells. If humans are infected with SARS-CoV-2, these antibodies will fight against SARS-CoV-2 (Fig. 4c). For example, Novavax is a recombinant protein subunit vaccine that was produced with SARS-CoV-2 glycoproteins and matrix-M adjuvant (derived from saponin). A Phase 3 study conducted in the United States and Mexico using Novavax exhibited 90.4% effectiveness in people (aged 18 and 84 years) [113]. Recently, Phase 3 clinical trials of Novavax's in the UK showed immunization effectiveness against COVID-19 of 89% when using the saponin-based Matrix-M adjuvant. The neutralizing antibody titer for NVX-CoV2373 peaked between 3.5 and 6 months following vaccination and was higher than Ad26.CoV2.S but equivalent to the mRNA vaccines, BNT162b2 and mRNA-1273 [92]. Although none are currently in use, more than 60% of vaccines being studied use the protein subunit technique [114]. The most frequent adverse effects of NVX-CoV2373, which last for two to three days, are headache, weariness, and malaise. Systemic side effects include myocarditis and pericarditis.

### 7.7 COVID-19: booster shots

A booster shot is a dose of vaccine given after receiving the primary vaccinations. Immunity against the virus can decrease over time after receiving the prior vaccinations, and a booster shot can help to boost the immune and leads to fighting against viruses effectively. Since the Omicron strain was identified, various countries have made significant changes to their vaccination program, including the recommendation of a third injection of boosting vaccine dose in large populations to reduce some adverse effects (Table 4) [115]. With immunity-boosting injections, COVID hospital admissions could be reduced. If the protection does fade off more quickly than expected, booster shots may be required every 6–12 months to prevent an increase in hospitalizations and mortality. The unvaccinated populations also significantly lead the way in arising new SARS-CoV-2 variants, including Omicron. Researchers also firmly say that differences in vaccination rates between countries would not end the pandemic and emerging SARS-CoV-2 variants [116]. To prevent any adverse effects, the emerging Omicron variants transmission should be stopped using booster vaccines [117]. However, many countries started to vaccinate with booster doses in full gear after the appearance of the Omicron variant. In the meantime, Johnson & Johnson, Moderna, and Pfizer reported that their vaccines are still effective against SARS-CoV-2 [118]. Moreover, Moderna reported that they are checking the vaccine's efficacy against the Omicron variant [119,120] Nevertheless, more research is still needed to understand SARS-CoV-2 transmissibility and the severity of variants.

**Table 4:** **Vaccines developed against SARS-CoV-2 strains**. The details on type of vaccine, product, total amount of mRNA per dose, age, doses for normal and other people with health issues, including the details on booster doses as per National Institute of Health (NIH) and World Health Organisation (WHO) recommendations.

S. No.	Type of vaccine	Product	Total amount of mRNA per dose	Age	For most people		For moderately or severely immunocompromised people		Booster doses
					Doses/mL	Duration between doses	Doses/mL	Duration between doses	
1.	mRNA	Moderna (Monovalent)	25 mcg per 0.25 mL	6 months - 5 years	2 doses of 0.25 mL	4-8 weeks	3/0.25 mL	4 weeks	–
		Moderna (Monovalent)	50 mcg per 0.5 mL	6-11 years	2 doses of 0.5 mL	4-8 weeks	3/0.5 mL	4 weeks	–
		Moderna (Bivalent)	25 mcg per 0.25 mL	6-11 years	3^a^	8 weeks	4	8 weeks	0.25 mL
		Moderna (Monovalent)	100 mcg per 0.5 mL	12-17 years	2 doses of 0.5 mL	4-8 weeks	3/0.5 mL	4 weeks	–
		Moderna (Bivalent)	50 mcg per 0.5 mL	12-17 years	3^a^	8 weeks	4	8 weeks	0.5 mL
		Pfizer-BioNTech (Monovalent)	3 mcg per 0.2 mL	6 months - 4 years	3 doses of 0.2 mL	3-8 weeks	3/0.2 mL	3-8 weeks	–
		Pfizer-BioNTech (Monovalent)	10 mcg per 0.2 mL	5-11 years	2 doses of 0.2 mL	3-8 weeks	3/0.2 mL	3-4 weeks	–
		Pfizer-BioNTech (Bivalent)	10 mcg per 0.2 mL	5-11 years	3^a^	8 weeks	4	8 weeks	0.2 mL
		Pfizer-BioNTech (Monovalent)	30 mcg per 0.3 mL	12-17 years	2 doses of 0.3 mL	3-8 weeks	3/0.3 mL	3-4 weeks	–
		Pfizer-BioNTech (Bivalent)	30 mcg per 0.3 mL	12-17 years	3^a^	8 weeks	4	8 weeks	0.3 mL
		Moderna (Monovalent)	100 mcg per 0.5 mL	18 years and above	2 doses of 0.5 mL	4-8 weeks	3/0.5 mL	4 weeks	–
		Moderna (Bivalent)	50 mcg per 0.5 mL	18 years and above	3^a^	8 weeks	4	8 weeks	0.5 mL
		Pfizer-BioNTech (Monovalent)	30 mcg per 0.3 mL	18 years and above	2 doses of 0.3 mL	3-8 weeks	3/0.3 mL	3-4 weeks	–
		Pfizer-BioNTech (Bivalent)	30 mcg per 0.3 mL	18 years and above	3^a^	8 weeks	4	8 weeks	0.3 mL
2.	Protein subunit vaccine	Novavax (Monovalent)	–	12 years and above	2^a^	3-8 weeks	2	3 weeks	–
		Novavax (Monovalent)	–	18 years and above	2^a^	3-8 weeks	2	3 weeks	–
3.	Adenovirus vector vaccine	Janssen (Monovalent)	Janssen COVID-19 vaccine is authorized for use in certain limited situations due to safety considerations						

a Dosage mL not revealed.

### 7.8 Effectiveness of homologous and heterogeneous vaccination against SARS-CoV-2

Several countries worldwide started to evaluate the effectiveness of homologous and heterogeneous vaccination against SARS-CoV-2, including their variants. For example, Jara et al. (2022_ group have assessed the efficiency of AZD1222 and BNT162b2 with the CoronaVac injected people. They selected persons (including single doses) who administrated vaccines from Feb 2021 to Nov 2021. Totally, 11,174,257 peoples were eligible for this study, in those 4, 127, 546 people administrated CoronaVac double doses and also received a booster dose. In this study, 46.5%, 48.9%, and 4.5% administered an AZD1222, BNT162b2, and homologous booster with CoronaVac, respectively. They calculated the effectiveness of the vaccines using a weighted stratified Cox model for a three-dose schedule with CoronaVac. The results showed 86.3%, 96.1%, and 97.7% (hospitalization), 92.2%, 96.2%, and 9897% (ICU admission), 86.7%, 96.8%, and 98.1% (death) for homologous CoronaVac booster, BNT162b2 booster, AZD1222 booster [121]. These results revealed that homologous and heterogeneous CoronaVac booster aids in a high level of protection against SARS-CoV-2 [121]. Similarly, the effectiveness of homologous and heterogeneous boosting vaccines with BNT162b2 booster was compared. This study revealed that homologous and heterogeneous CoronaVac booster aids in a high level of protection against SARS-CoV-2 than the BNT162B2 [122]. However, this homologous and heterogeneous vaccination also could play a potential role in treating COVID-19 patients.

Researchers across the globe are vigorously working around the clock to develop a effective vaccines against COVID-19. As of January 31, 2023, there are more than 200 potential vaccines are in various stages of development. While some are in clinical trials and some are approved by the WHO (Table 4, Table 5). Acquisition of genetic mutations in SARS-CoV-2 also created a serious threat among the people world. Therefore, the developed vaccines’ effectiveness against SARS-CoV-2 variants including Omicron has been questioned. However, scientists started to check the efficiency of vaccines against the emerging SARS-CoV-2 variants Table 6, Table 7(. Science has overcome many pandemics and diseases like flu, polio, malaria, etc by developing effective vaccines. Similarly, science will overcome COVID-19 soon .

**Table 5:** **Vaccines approved after phase 3 trials**. The details on name of the vaccine, vaccine type, vaccine developers, vaccine effectiveness, dose, storing temperature with their origin country are listed.

S. No.	Name	Vaccine type	Vaccine developers	Efficacy	Dose	Storage	Origin (Country)
1.	Comirnaty (BNT162b2)	mRNA-based vaccine	Pfizer, BioNTech, and Fosun Pharma	95%	2 doses (Muscle injection)	−94 °F (−70 °C)	Multinational
2.	Moderna	COVID-19 mRNA-based Vaccine (mRNA-1273)	Moderna, BARDA, NIAID	94.5%	2 doses (Muscle injection)	6 months at −4 °F	US
3.	AstraZeneca (AZD1222)	Adenovirus vaccine	BARDA	62% to 90%	2 doses (Muscle injection)	6 months at −4 °F	UK
4.	Convidecia (Ad5-nCoV)	Recombinant vaccine	CanSino Biologics	–	1 dose (Muscle injection)	Refrigerator	China
5.	Covaxin (BBV152 A, B, C)	Inactivated vaccine	Bharat Biotech, ICMR	–	2 doses (Muscle injection)	Room temperature	India
6.	Sputnik V	Non-replicating viral vector	Acellena Contract Drug Research and Development	91.4%	2 doses (Muscle injection)	Freezer storage	Russia
7.	CoronaVac (PiCoVacc)	Inactivated vaccine	National Pharmaceutical Group	50.38%	2 doses (Muscle injection)	Refrigerator	China
8.	BBIBP	Inactivated vaccine	Beijing Institute of Biological Products	79.34%	2 doses (Muscle injection)	–	China
9.	EpiVac Corona	Peptide vaccine	Federal Budgetary Research Institution State Research Center of Virology and Biotechnology	–	2 doses (Muscle injection)	Refrigerator	Russia
10.	Comirnaty (BNT162b2)	mRNA-based vaccine	Pfizer, BioNTech; F osun Pharma	95%	2 doses (Muscle injection)	−94 °F (−70 °C)	Multinational

**Table 6:** **Effectiveness of vaccines against variant of concerns of SARS-CoVv-2 strains**. The details on name of the vaccines and variants of SARS-CoV-2 concern strains with their references.

Vaccines	Variant of concerns				
	B.1.	B.1.1.7	B.1.351	B.1617	P.1
AstraZeneca	66.7% VE [123]	70.4% VE [124]	No protection [125]	59.8% VE [126]	NA
Johnson & Johnson	66.7% VE [127]	NA	64% VE	NA	NA
Pfizer	95% VE [127]	93.4% VE [[126], [127]]	75% VE [127]	87.9% VE [[125], [126], [127], [128]]	Reduction in neutralizing activity [[127], [129]]
Moderna (ADZ1222)	94% VE [127][127]	No significant reduction in neutralizing activity [29]	Significant reduction neutralizing activity [29]	Reduction in neutralizing activity [128]	Reduction in neutralizing activity [127]
Sinopharm	79% VE [127]	Faintly declines the neutralizing activity of vaccine era [130]	Resistance to post vaccination sera with partial reduction in neutralizing activity [130]	NA	NA
Sinovac Biotech	50.7% VE [131]	NA	NA	NA	49.6% VE [131]
BNT162b2	NA	90% VE [132]	75% VE [132]	Reduction in neutralizing activity [[128], [133], [134]][128,133,134]	Neutralizing activity of vaccine era [135]
Ad26.CoV2.S	NA	72% VE [136]	64% VE [136]	Reduction in neutralizing activity [[137], [138]]	64% VE [136]
ChAdOx1 nCoV-19	NA	74% VE [139]	81.5% VE [124][124]	67% VE [139]	81.5% VE [124]
NVX-CoV2373	NA	89.3% VE	49.4% VE [140]	49.4% VE [135]	NA
Covaxin	NA	No reduction [130]	NA	Resistance to neutralizing antibodies [141]	50.4% (symptomatic person) and 78% (asymptomatic person) [142]
Sputnik V	NA	No reduction [143]	Reduction in neutralizing activity [143]	Reduction in neutralizing activity [144]	Reduction in neutralizing activity [145]
BBIBP-CorV	NA	No reduction [143]	No reduction [[130], [143]]	NA	NA
Corona Vac	NA	NA	NA	NA	Reduction in neutralizing activity [[140], [146]]

Abbreviations: NA-not available.

**Table 7:** **Effectiveness of vaccines against variant of interests of SARS-CoV-2 strains**. The details on name of the vaccines and variants of SARS-CoV-2 interest strains with their references.

Vaccines	Variant of interest							
	Epsilon	Zeta	Eta	Theta	Iota	Kappa	Lambda	Mu
Moderna	Neutralizing antibodies [147]	NA	NA	NA	94-95% [148]	NA	Neutralizing antibodies [149]	94% [150]
BNT162b2	Neutralizing antibodies [151]	Neutralizing antibodies [152]	Neutralizing antibodies [153]	Neutralizing antibodies [146]	94-95% [148]	Neutralizing antibodies [153]	Neutralizing antibodies [123]	Neutralizing antibodies [154]
Ad26.CoV2.S	66% [155]	66% [155]	NA	NA	66% [155]	NA	NA	NA
ChAdOx1 nCoV-19	55-81% [[123], [148]]	55-81% [[123], [148]]	NA	NA	55-81% [123]	NA	55-81% [123]	NA
NVX-CoV2373	89% [156]	NA	NA	NA	89% [156]	NA	66% [148]	NA
Covaxin	NA	NA	NA	NA		78% [148]	NA	NA
Corona Vac	No difference in neutralizing antibodies [157]	Neutralizing antibodies [158]	NA	NA	Neutralizing antibodies [158]	NA	NA	NA

Abbreviations: NA-not available.

## 8 Limitations

This review literature has limitations. Primarily, we did not explain the significant entry molecules ACE2, TMPRSS2 and furin, CD147, and GRP78, the host cell receptor adopted by the virus to enter humans. Second, we did not discuss the detection methods such as Clustered regularly interspaced short-palindromic repeats (CRISPR) based technology (discussed in our previous article [159]), sequence-based methods, protein-based detection methods, Viral Transcript Usage Sensor (VTUS), point of care (POC), and X-ray based detections in the manuscript. However, this study aims to provide the dynamic nature of SARS-CoV-2 transmission, SARS-CoV-2 variants (a variant of concern and interest), antiviral drugs, and vaccines utilized against SARS-CoV-2 at a glance. We hope this study will enable the researcher to gain knowledge on SARS-CoV-2 variants, antiviral drugs, and vaccines, which will also pave the way to identify efficient novel vaccines or medicines against forthcoming SARS-CoV-2 strains.

## 9 Conclusion

The SARS-CoV-2 outbreak has challenged the world's public health, medical, and economic systems. More novel variants of SARS-CoV-2 are also expected to originate in the future. Therefore, efforts should be made to develop wide-ranging measures to prevent future outbursts of zoonotic origin. We believe this article will provide essential and up-to-date information about SARS-CoV-2 variants, antiviral drugs, and vaccines used to fight it. This article will help healthcare professionals and researchers and generate awareness about the SARS-CoV-2 diseases. However, further studies are needed to challenge and combat the forthcoming SARS-CoV-2 variants.

## Ethics approval

Not applicable.

## Availability of data and materials

Not applicable to this article.

## Consent to participate

Not applicable.

## Consent for publication

All the authors have approved the manuscript for publication.

## Author contribution statement

Stanislaus Antony Ceasar, Varghese Edwin Hillary: Conceived and designed the experiment; Analyzed and interpreted the data; Wrote the paper.

## Funding statement

This research did not receive any specific grant from funding agencies in the public, commercial, or not-for-profit sectors.

## Data availability statement

No data was used for the research described in the article.

## Declaration of interest’s statement

The authors declare no competing interests.
